# Cardiac arrhythmias classification using photoplethysmography database

**DOI:** 10.1038/s41598-024-53142-9

**Published:** 2024-02-09

**Authors:** Qasem Qananwah, Marwa Ababneh, Ahmad Dagamseh

**Affiliations:** 1 Department of Biomedical Systems and Informatics Engineering, Hijjawi Faculty for Engineering Technology, Yarmouk University, P.O.Box 21163, Irbid, Jordan; 2 Department of Computer Engineering, Hijjawi Faculty for Engineering Technology, Yarmouk University, Irbid, Jordan; 3 Department of Electronics Engineering, Hijjawi Faculty for Engineering Technology, Yarmouk University, Irbid, Jordan

**Keywords:** Computational biology and bioinformatics, Medical research, Engineering

## Abstract

Worldwide, Cardiovascular Diseases (CVDs) are the leading cause of death. Patients at high cardiovascular risk require long-term follow-up for early CVDs detection. Generally, cardiac arrhythmia detection through the electrocardiogram (ECG) signal has been the basis of many studies. This technique does not provide sufficient information in addition to a high false alarm potential. In addition, the electrodes used to record the ECG signal are not suitable for long-term monitoring. Recently, the photoplethysmogram (PPG) signal has attracted great interest among scientists as it provides a non-invasive, inexpensive, and convenient source of information related to cardiac activity. In this paper, the PPG signal (online database Physio Net Challenge 2015) is used to classify different cardiac arrhythmias, namely, tachycardia, bradycardia, ventricular tachycardia, and ventricular flutter/fibrillation. The PPG signals are pre-processed and analyzed utilizing various signal-processing techniques to eliminate noise and artifacts, which forms a stage of signal preparation prior to the feature extraction process. A set of 41 PPG features is used for cardiac arrhythmias' classification through the application of four machine-learning techniques, namely, Decision Trees (DT), Support Vector Machines (SVM), K-Nearest Neighbors (KNNs), and Ensembles. Principal Component Analysis (PCA) technique is used for dimensionality reduction and feature extraction while preserving the most important information in the data. The results show a high-throughput evaluation with an accuracy of 98.4% for the KNN technique with a sensitivity of 98.3%, 95%, 96.8%, and 99.7% for bradycardia, tachycardia, ventricular flutter/fibrillation, and ventricular tachycardia, respectively. The outcomes of this work provide a tool to correlate the properties of the PPG signal with cardiac arrhythmias and thus the early diagnosis and treatment of CVDs.

## Introduction

Worldwide, Cardiovascular Diseases (CVDs) are the number one cause of death^1,2^. Cardiac arrhythmia is caused by an abnormality in the sequence of the electrical impulses that control the heartbeat, resulting in an irregular heartbeat. This leads to low cardiac output and poor blood flow to the rest of the body. This type of arrhythmia mainly originates in the atria or ventricles.

Generally, there are different types of cardiac arrhythmias such as; ventricular fibrillation, atrial fibrillation, tachycardia, bradycardia, etc…^3,4^. Tachycardia occurs when the electrical impulses propagate rapidly along the heart, so the heart rate (HR) increases and the heartbeat fast, while Bradycardia occurs when the HR is low, resulting in a decreased cardiac output. Atrial fibrillation occurs when no visible p-wave precedes the QRS complex in an electrocardiogram (ECG). Finally, ventricular fibrillation occurs when the ventricles are pre-excited by the contraction of the atria. Therefore, cardiac arrhythmias monitoring is very important to prevent heart attacks.

The ECG is the common diagnostic method for cardiac arrhythmias monitoring since it is tracing the electrical activity of the heart^5,6^. Several research groups have been working to improve the performance of arrhythmias detection through ECG pre-processing, feature extraction, feature selection, and arrhythmias classification using machine learning or deep learning techniques^6–13^.

Lin and Yang developed a classifier to identify cardiac arrhythmias using standardized ECG morphological features from the MIT-BIH database^6^. The testing results showed that the presented techniques improved the classification accuracy to 93% and the sensitivity to identify atrial premature was 21%. Yu and Chen proposed an ECG beat classification technique utilizing a two-level wavelet transform and probabilistic neural network^8^. With eleven features, they were able to achieve a classification accuracy of 99.6% for six ECG beats types. Arif et al. provided a beat classification system using discrete wavelet transform utilizing ECG signal^9^. They achieved a 99.5% classification accuracy for six types of ECG rhythms utilizing an NN classifier with 11 features. Acharya et al. proposed a computer-aided diagnostic system to automatically detect different ECG segments using the convolutional neural network (CNN) technique with an accuracy of 92.5%^10^. Kiranyaz et al. presented an ECG-based classification system with the MIT-BIH database using convolutional neural networks^11^. They claim that their system has achieved a superior classification performance for the detection of ventricular ectopic beats and supraventricular ectopic beats with 99% accuracy. Zhang and Luo developed a multi-lead fused classification scheme to improve the arrhythmias classification using the MIT-BIH database^12^. The classification accuracy was 87.8% with sensitivity for normal, supraventricular ectopic, ventricular ectopic, and fusion of ventricular of 88.6%, 74.3%, 88%, and 73.4%, respectively. Desai et al. presented a study on the utilization of ECG signal in the classification of cardiac arrhythmias, namely; Atrial Fibrillation (A-Fib), Atrial Flutter (AFL), Ventricular Fibrillation (V-Fib), and Normal Sinus Rhythm (NSR) using ensemble classifiers^13^. The results demonstrate that the Rotation Forest (ROF) ensemble classifier achieves the highest accuracy of 98.37% compared to Decision Tree (DT), Random Forest (RAF). Li et al. discusses multi labels classification of heart arrhythmias utilizing the highly dimensional ECG featuers^14^. They proposed a design of a criterion of ECG features based on kernelized fuzzy rough sets for optimal feature subset and subsequently, to enhance the precision of arrhythmia identification. Six metrics were utilized to evluate the performance of the proposed technique.

However the huge number of research that has been performed using the ECG signal for arrhythmias detection, it is subjected to a high likelihood of false alarms as well as the complex electrode connections, making it an undesirable technique for long-term arrhythmia monitoring. Alternatively, Photoplethysmography (PPG) has emerged as a non-invasive, simple, inexpensive, affordable, and convenient technique capable of monitoring hemodynamic parameters. The PPG signal is measured with the optical technique and reflects the volume of blood change in the arteries^15,16^. Additionally, due to the detection principle of the PPG signal in utilizing the optical technique, it is suitable and widely accepted for integration with wearable devices. Thereby, it is considered the preferable choice as an alternative to the ECG technique for cardiac arrhythmias monitoring^17,18^. Limited research has been performed for multiple arrhythmias detection using the MIMIC/PhysioNet online PPG signal database. Chong et al. proposed an arrhythmia classification technique using the PPG signal for smartphones. Four arrhythmias were considered, normal sinus rhythm (NSR), atrial fibrillation (AF), premature ventricular contractions (PVC), and premature atrial contraction (PAC). The results showed that the system could classify the PAC and PVC with an accuracy of 97% and 100%, respectively. Sološenko et al. Proposed a PPG-based system for the detection of bradycardia and tachycardia arrhythmias using a dual-branch convolutional neural network (CNN)^19^. The PhysioNet/CinC 2015 Challenge Database was used as input for the performance evaluation of the system. The results showed that the system could detect bradycardia and tachycardia arrhythmias with an accuracy of 98% and 77%, respectively. In another article, Sološenko et al. presented a method for detecting premature ventricular contractions (PVCs) using the PhysioNet PPG signal databases for training and testing^20^. The artificial neural network was used as a classifier with an accuracy and sensitivity of 92% and 93%, respectively. Cheng et al. developed a machine-learning model for atrial fibrillation detection using PPG signals from online databases^21^. They utilized deep learning techniques with time–frequency analysis. The results showed that the developed system is able to detect atrial fibrillation with an accuracy of 98%. Feed-forward artificial neural network was utilized by Neha et al. to classify four different types of arrhythmias (i.e. normal, PVC, atrial flutter (AFI), and Sinus Tachycardia (ST))^22^. The feature extraction process was performed utilizing the dynamic time warping technique applied to the PPG signal from the PhysioNet MIMIC-II database. The results showed that the proposed model could classify multiple types of arrhythmias with 96% accuracy. In another work, Neha et al. utilized a set of morphological features for multiple arrhythmias classification with a statistical learning-based approach^23^. The results showed that the proposed detection technique has 94% detection accuracy. Clifford designed an algorithm to estimate the heart rate and detect PVC on the PhysioNet MIMIC PPG signal database^24^. With two morphological features, they were able to a PVC detection accuracy of 99%. Although some previous studies have shown good performance, they are not exhaustive in that they are limited to one or two types of arrhythmias, restricted to one classification technique, or hindered by inter-waveform alignment problems. Additionally, the features used in the detection stage were limited to the utilization of either time-domain or frequency-domain features. This study exclusively utilized the PPG signal to identify arrhythmias. The detection stage employed a variety of features, including morphological, statistical, and frequency domain features, to investigate four types of cardiac arrhythmias, namely bradycardia, tachycardia, ventricular fibrillation or flutter (VFB), and ventricular tachycardia (VTA). The classification performance was assessed in conjunction with significant features and employing various machine learning techniques, including Support Vector Machine (SVM), K-Nearest Neighbor (KNN), Decision Trees (DT), and Ensemble techniques. This research contributes to the advancement of portable and wearable devices that can be used for the diagnosis and monitoring of various types of cardiac arrhythmias^25^.

## Materials and methods

In this research, the PPG waveforms obtained from publicly accessible databases (specifically, PhysioNet/Computing in Cardiology Challenge 2015) were utilized to train, validate, and test classification models. These databases contain over 750 raw waveforms from bedside monitors in intensive care units (ICUs), each consisting of two ECG leads and at least one pulsatile waveform of either PPG or arterial blood pressure (ABP) waveform for a duration of 5 min. Each waveform is associated with an alarm that indicates either a true or false arrhythmia event. For this study, only PPG waveforms with true alarms were considered. The PPG signals utilized in this study are raw signals, imitating the dynamic scenarios of signal acquisition. The preprocessing step was used to prepare the data for feature extraction as shown in Fig. 2. The preprocessing includes filtration of the signal utilizing a bandpass filter between (0.05 Hz to 30 Hz). Furthermore, the signal smoothing using moving average filter, then, the baseline wandering was removed using wavelet transform followed with signal normalization. Each waveform was segmented into 10-s intervals. The total number of arrhythmia cases was 55, comprising 12 cases of Tachycardia, 20 cases of VT, 5 cases of VF, and 18 cases of Bradycardia, in addition to healthy cases. The data was split using the cross-validation technique, and the k-value was set to 10. This k-value is very common in the field of machine learning, as it has been shown to give a low test-error rate with less bias and small variance. The arrhythmias investigated in this study were bradycardia, tachycardia, ventricular fibrillation or flutter (VFB), and ventricular tachycardia (VTA).

**Figure 1:**
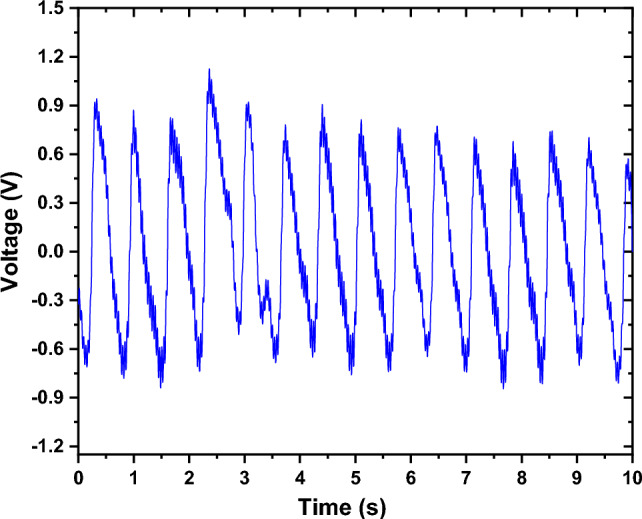
An example of raw PPG signal.

**Figure 2:**
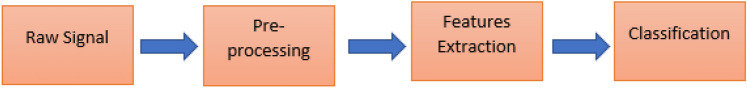
A block diagram outlining the pre-processing procedure for PPG signals.

Figure 1 shows an example of a raw PPG signal. The PPG waveforms were obtained and subsequently subjected to offline analysis and pre-processing. The initial processing steps encompassed filtration, smoothing, wavelet transformation (to eliminate baseline wandering), and normalization. Figure 2 illustrates a block diagram of the pre-processing procedure executed on the PPG waveforms.

**Figure 3:**
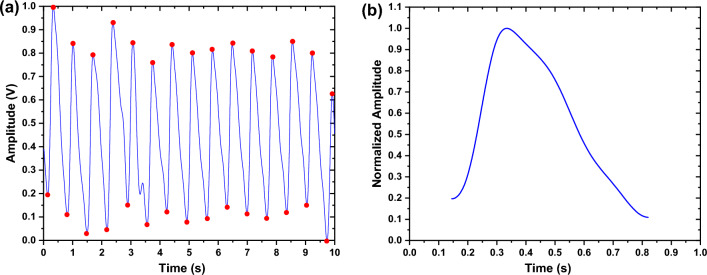
Example for (**a**) Valleys' detection, and (**b**) one segment of the PPG signal.

The preprocessing stage was succeeded by the feature extraction process, which aimed to analyze the PPG signals. Feature extraction is a crucial process that is necessary to identify the significant features in the signals that are pertinent to the conditions and arrhythmias being investigated. Prior to feature extraction, peaks and valleys detection, notch localization (i.e., identifying the start and end of the notch), and segmentation were carried out for each signal. The detection of valleys in the PPG signal aids in determining the start and end of each pulse, which is a critical step in segmentation (i.e., the process that precedes feature determination for each segment, as depicted in Fig. 3a). The segmentation process is accomplished by dividing the signals into their corresponding pulses by utilizing the valleys (as illustrated in Fig. 3b).

**Figure 4:**
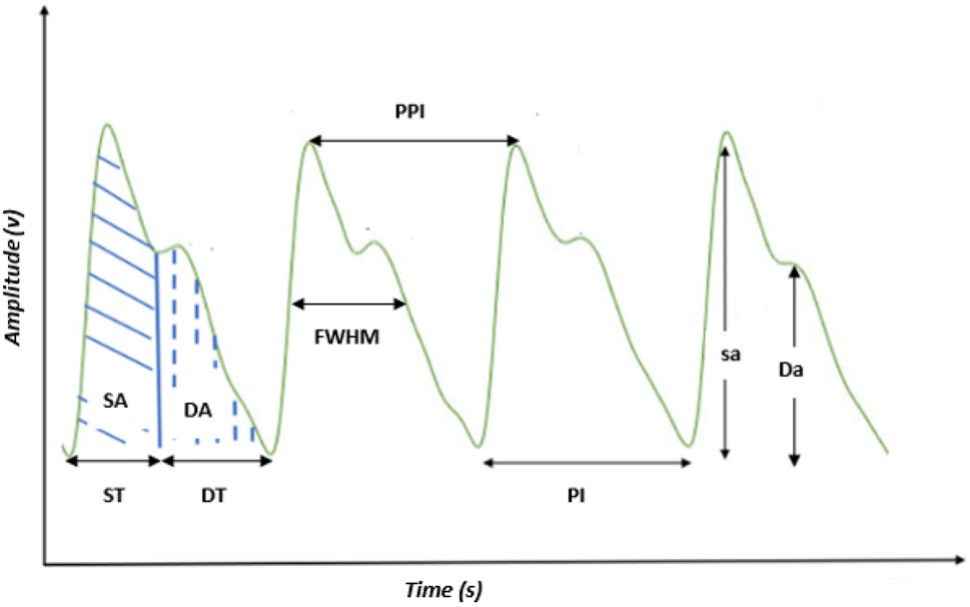
The principles of the morphological features of the PPG signal.

The PPG signal is capable of encoding information pertaining to cardiac arrhythmias, given that these arrhythmias have an impact on the characteristics of the PPG waveform. This study involved the extraction of 41 features from the PPG signal, encompassing both morphological and frequency domain features. The mean and standard deviation were also calculated for all features. Figure 4 provides a visual representation of the principles underlying the morphological features and Table 1 shows example of some PPG features utilized in this study.

**Table 1:** Example of some PPG features utilized in the current study. The features' definitions are shown in Fig. 4.

Feature name	Feature abbreviation
Systolic amplitude	sa
Diastolic amplitude	Da
Systolic area	SA
Diastolic area	DA
Systolic time	St
Diastolic time	Dt
Pulse Interval	PI
Peak-to-Peak Interval	PPI
Full Width Half Maximum	FWHM

The phase of feature extraction is the most critical aspect of the classification process, as the reliability and precision of this step are pivotal in achieving optimal classifier performance^26^. It is imperative to identify the most representative and significant set of features to ensure robustness and accuracy. Subsequently, the classification stage is executed using Machine Learning (ML) techniques.

In the classification of cardiac arrhythmias, preprocessed PPG waveforms obtained from the online database, PhysioNet Challenge 2015, were utilized to extract features that represent the embedded information of each waveform. These features were then employed in various classification models to categorize different types of cardiac arrhythmias. The models were optimized to achieve high classification accuracy.

Various metrics were employed to assess the effectiveness of the classifiers for cardiac arrhythmias, including accuracy, sensitivity, specificity, and precision where:

1 $$Accuarcy=\frac{TP+TN}{TP+TN+FP+FN}$$

- Accuracy refers to the proportion of correct predictions to the total number of predictions made.
  2 $$Sensitivity=\frac{TP}{TP+FN}$$
- Sensitivity refers to the rate of true positives, which is determined by dividing the number of accurate positive predictions by the total number of positive outcomes.
  3 $$Specificity= \frac{TN}{TN+FP}$$
- The concept of specificity pertains to the accurate identification of negative outcomes. It is calculated by dividing the number of true negative predictions by the total number of negative outcomes.
  4 $$Pricision=\frac{TP}{TP+FP}$$
- Precision refers to the proportion of accurate positive predictions to the overall number of positive predictions.

In the context of predictive modeling, TP denotes the count of accurate predictions made by the model for the positive class, while TN signifies the count of accurate predictions made for the negative class. Conversely, FP refers to the count of inaccurate predictions made for the positive class, and FN refers to the count of inaccurate predictions made for the negative class.

### Ethical approval

The authors affirm that all activities carried out in research involving human subjects were conducted in compliance with the ethical guidelines set forth by the institutional and/or national research committee, as well as the 1964 Helsinki Declaration and its subsequent revisions or equivalent ethical standards. Furthermore, the authors assert that no animals were utilized in the study.

## Results and discussions

The features were employed to classify various arrhythmias, such as Bradycardia, Tachycardia, Ventricular Tachycardia (VT), and Ventricular Fibrillation (VF). The outcomes of heart arrhythmias classification, based on 41 features as an input vector, are presented in Table 2. It is noteworthy to mention that several studies^27–32^ have utilized the PhysioNet challenge database to explore and mitigate erroneous arrhythmia alerts during uninterrupted patient surveillance. Upon examination of the True Positive rate for each class in terms of classification sensitivities, the outcomes were found to be consistent with those documented in previous literature^27–33^.

**Table 2:** Cardiac arrhythmias classification results using 41 PPG features.

Classifier	Classification report				
	Arrhythmia	Sensitivity (%)	Specificity (%)	Precision (%)	Accuracy (%)
DT	Brady	98.5%	93.3%	99.8%	93.1%
	Tachy	85%	85.8%	96.8%	
	VF	82%	85.2%	98.1%	
	VT	96.6%	95.7%	93.3%	
KNN	Brady	98.3%	99.3%	100%	97.3%
	Tachy	94%	96%	100%	
	VF	90.5%	99.7%	98.3%	
	VT	99.4%	95%	96.7%	
SVM	Brady	100%	100%	100%	96.7%
	Tachy	91%	99.3%	96.8%	
	VF	92.1%	99.3%	95.1%	
	VT	98.8%	94.5%	96.4%	
Ensemble	Brady	98.3%	100%	100%	97.4%
	Tachy	93%	100%	100%	
	VF	92.1%	99.6%	96.7%	
	VT	99.7%	94.6%	96.7%	

The results indicate that the Ensemble and KNN models exhibited the most efficient classification performance, achieving accuracies of 97.4% and 97.3%, respectively, among all the machine-learning techniques employed. It is noteworthy that the Ensemble results in overall classification algorithms demonstrate an enhanced prediction performance compared to any individual algorithm. The sensitivity of bradycardia was high across all machine learning models, accurately classifying instances of bradycardia. This can be attributed to the straightforward nature of certain features related to pulse interval values in bradycardia.

In SVM, KNN, and Ensemble, there exists an inverse correlation between the sensitivity and specificity for VT, VF, and Tachycardia. The sensitivity for VF and Tachycardia is low due to the dominance of the positive class, while the specificity for VT is low, indicating a low true negative value and a high false negative value. The high sensitivity of VT is attributed to the large number of VT samples in the dataset. Sensitivity and precision are interrelated as they are both utilized in the calculation of true positives. However, precision is higher as a false positive is lower than a false negative, whereas sensitivity depends on the false negative.

When evaluating the sensitivity of each class, it was found that the classification outcomes achieved through the use of just the PPG signal were superior to those reported in previous studies^27^^–^^32^, particularly for VT and VF, as demonstrated in Table 3. In a study conducted by the authors of reference ^33^, the same Physio Net Challenge 2015 database was utilized to classify cardiac arrhythmias using the PPG signal, resulting in an overall accuracy of 93%, which is lower than the accuracy achieved in this study. As previously mentioned, the exclusive use of the PPG signal has the potential to significantly enhance the detection and monitoring of cardiac arrhythmias, taking advantage of its noninvasive nature. Table 3 provides a summary of the results obtained in the literature for the classification of cardiac arrhythmias using bio-signals, in comparison to the findings of this study.

**Table 3:** The classification results of cardiac arrhythmias of the current study using the Physio Net Challenge 2015 database compared with the literature.

Reference	Signal type	Sensitivity			
		Brady	Tachy	VF	VT
Plesinger et al.^27^	ECG, PPG, ABP	100%	97%	67%	85%
Fallet et al.^28^	ECG, PPG, ABP	96%	96%	83%	93%
Antink et al.^29^	ECG, PPG, ABP	100%	100%	67%	90%
Eerikanen et al.^30^	ECG, PPG, ABP	96%	99%	75%	84%
Kalidas et al.^31^	ECG, PPG	100%	100%	100%	84%
Caballero et al.^32^	PPG, ABP	95%	97%	89%	49%
Paradkar et al.^33^	PPG	88.8%	97.6%	50%	88.1%
Current study*	PPG	98.3%	93%	92.1%	99.7%

*Using the Ensemble technique.

**Figure 5:**
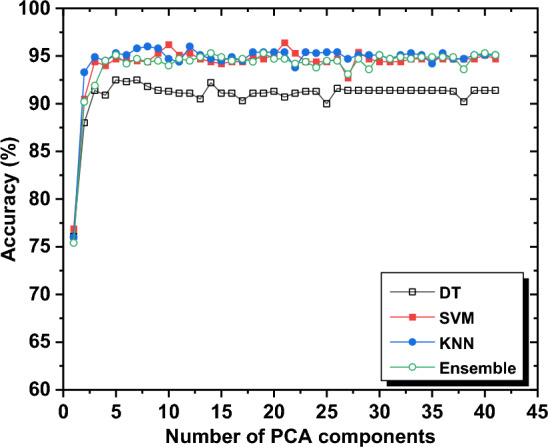
The classification accuracy for different techniques with different PCA components.

The Principal Component Analysis (PCA) technique was used to reduce the dimension of the input vector and evaluate the accuracy of different machine learning algorithms versus the number of PCA components. Figure 5 shows the accuracy of different classification techniques as a function of the number of PCA components. The results show that the accuracy did not deteriorate significantly when the number of features in the input vector of the classifier was reduced. This could be beneficial in reducing the number of features without significantly affecting accuracy. The DT algorithm achieved the highest accuracy of 92.5% with only seven PCA components. Conversely, the SVM and KNN algorithms achieved their highest accuracy of 96.4% and 96%, respectively, using twenty-one (for the SVM) and eight PCA components (for the KNN). The Ensemble technique achieved its highest accuracy of 95.3% with nineteen PCA components. Increasing the number of PCA components beyond the specified number did not result in any performance improvement for the classification techniques.

It can be observed that, with the exception of the DT algorithm (both pre- and post-PCA), all classification techniques may be deemed suitable for constructing the classification model. The DT algorithm's inferior accuracy may be attributed to the potential for overfitting, resulting in a complex model that fails to generalize data effectively. Additionally, if the number of waveforms is not evenly distributed across all classes, the DT algorithm may produce a biased model.

The process of selecting features was carried out through the utilization of PCA on the complete datasets. The purpose of the PCA was to examine the level of correlation between each extracted feature and the first principal components, identify the most significant features, and eliminate the irrelevant ones. The 41 features of each signal were transformed into a new set of variables, referred to as principal components, which are uncorrelated and arranged in order of significance, with the first component retaining the highest explained variance among all the original features. The explained variance denotes the information explained by a specific principal component.

**Figure 6:**
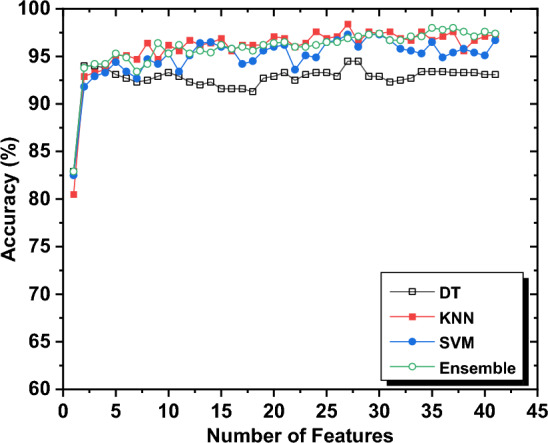
The accuracy of different classification techniques with the number of features.

The process of selecting features was carried out iteratively, as previously described. A summary of the performance of four machine-learning techniques, as the number of features increased, is presented in Fig. 6. The results indicate that the selected features have marginally enhanced the classification performance. Given the similarities between Tachycardia, VF, and VT, it was necessary to exploit the dependent features for each of them by utilizing more features. The classification outcomes and the optimal features selected are summarized in Table 4.

**Table 4:** Classification results obtained using the selected features.

Classifier	Classification report				
	Arrhythmia	Sensitivity	Specificity	Precision	Accuracy
KNN	Brady	98.3%	100%	100%	98.4%
	Tachy	95%	99.7%	98.9%	
	VF	96.8%	99.8%	98.3%	
	VT	99.7%	96.8%	97.9%	
SVM	Brady	98.3%	99.8%	98.3%	98%
	Tachy	91%	99.8%	98.9%	
	VF	96.8%	99.8%	98.3%	
	VT	100%	94.5%	96.5%	
Ensemble	Brady	100%	99.8%	98.3%	98%
	Tachy	93%	100%	100%	
	VF	93.7%	99.8%	98.3%	
	VT	100%	95.9%	97.3%	

**Figure 7:**
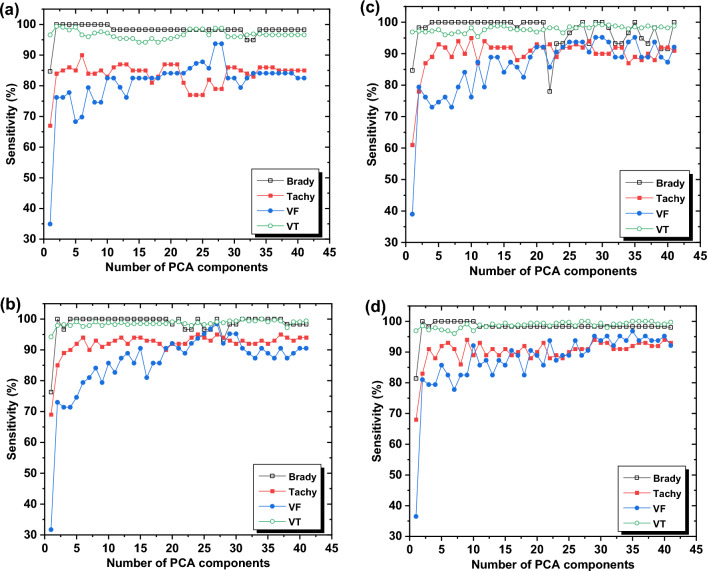
The classification sensitivity of different cardiac arrhythmias; namely AF, Bradycardia, Normal, and Tachycardia by the utilization of different machine learning techniques (**a**) DT, (**b**) SVM, (**c**) KNN, and (**d**) Ensemble.

The DT technique achieved a maximum accuracy of 94% and maintained a steady accuracy of 93.1%. On the other hand, other machine learning algorithms demonstrated high classification accuracy, with KNN achieving 98.4%, and Ensemble and SVM achieving both 98%. Figures 6 and 7 indicate that the accuracies and sensitivities of certain features increased while others decreased, which may be attributed to the hyper-parameters of the models. The inclusion of redundant features can lead to overfitting and mislead the algorithm's modeling. Additionally, the sensitivity of the classification, particularly for Tachycardia, can be affected by the inclusion of subjects with multi-arrhythmias.

Based on the findings presented in Fig. 7, it is evident that VT and bradycardia exhibit the highest sensitivity in comparison to other arrhythmias. This observation can be attributed to the larger number of VT samples as compared to Tachycardia and VF. It is noteworthy that the sensitivity, specificity, and precision of arrhythmias remain unaffected by data reduction, as they are influenced by data balancing, which ultimately determines the overall accuracy. The Ensemble classification, utilizing the same selected features as KNN and SVM, achieved an accuracy of 97.3%. Notably, there was no significant difference in accuracy between the fifteen features and thirteen features, with the latter achieving an accuracy of 98%.

The processing time of the training process is contingent upon the intricacy of the problem at hand and the complexity of the machine-learning algorithm employed. The training time for the DT, KNN, and SVM algorithms was found to be comparable. In contrast, the Ensemble technique involves the amalgamation of multiple learners, thereby necessitating a training time that is directly proportional to the number of learners. Consequently, the computational complexity of the Ensemble technique is higher, and it requires a longer training time than other machine learning algorithms. However, it is noteworthy that the number of learners is not contingent upon the number of features, and the optimizer is capable of identifying the optimal number and size that balances accuracy and speed.

The results show that the KNN, SVM, and Ensemble have similar levels of accuracy, as shown in Table 4. The classifier performs better when reducing the number of neighbors. In the case of a dataset with n data points, the KNN classifier utilizes the majority voting scheme to classify new points. On the other hand, only the nearest data points are selected in case the number of neighbors is limited. Increasing the number of data points could result in a decrease in the overall performance due to the increased probability of the inclusion of a data point from other classes.

## Conclusions

This study utilized a public online database of PPG signals (PhysioNet Challenge 2015) to classify different cardiac arrhythmias, such as bradycardia, tachycardia, ventricular tachycardia, and ventricular flutter/fibrillation. The PPG features were divided into three sets namely, training, validation, and testing. All PPG signals were preprocessed and features were extracted to be considered as input vectors for the classification model. Four machine learning techniques, namely decision tree (DT), k-nearest neighbors (KNN), support vector machine (SVM), and ensemble, were utilized in the classification process. The results demonstrated a high level of accuracy in classifying different arrhythmias, with an overall accuracy of 98.4% and sensitivities of 98.3%, 95%, 96.8%, and 99.7% for Bradycardia, Tachycardia, Ventricular Flutter/Fibrillation, and Ventricular Tachycardia, respectively. Furthermore, this study demonstrated that high accuracy in classifying cardiac arrhythmias could be achieved using only one type of bio-signal, specifically the PPG signal, and a limited number of features. This represents a significant advancement in the development of a monitoring system for detecting and classifying various cardiac arrhythmias.

## Data Availability

Data are publicly available online (i.e. PhysioNet/Computing in Cardiology Challenge 2015).

## References

[1] World Health Organization (WHO). Cardiovascular diseases (CVDs), (2021). [Online]. Available: https://www.who.int/news-room/fact-sheets/detail/cardiovascular-diseases-(cvds).

[2] L. A. Raffee *et al.* Prevalence, clinical characteristics, and risk among patients with ischemic heart disease in the young Jordanian population. *Open Access Emerg. Med.*, 389–397, (2020). 10.2147/OAEM.S272961 PMC7678703 33235526

[3] Antzelevitch C, Burashnikov A (2011). Overview of basic mechanisms of cardiac arrhythmia. Card. Electrophysiol. Clin..

[4] J. G. Webster. *Medical instrumentation: application and design*. Wiley, (2009).

[5] Yıldırım Ö, Pławiak P, Tan R-S, Acharya UR (2018). Arrhythmia detection using deep convolutional neural network with long duration ECG signals. Comput. Biol. Med..

[6] Lin C-C, Yang C-M (2014). Heartbeat classification using normalized RR intervals and morphological features. Math. Probl. Eng..

[7] E. J. da S. Luz, W. R. Schwartz, G. Cámara-Chávez, & D. Menotti. ECG-based heartbeat classification for arrhythmia detection: A survey. *Comput. Methods Programs Biomed.* 127, 144–164. 10.1016/j.cmpb.2015.12.008 (2016). 10.1016/j.cmpb.2015.12.008 26775139

[8] Yu SN, Chen YH (2007). Electrocardiogram beat classification based on wavelet transformation and probabilistic neural network. Pattern Recognit. Lett..

[9] Arif M (2008). Robust electrocardiogram (ECG) beat classification using discrete wavelet transform. Physiol. Meas..

[10] Acharya UR, Fujita H, Oh SL, Hagiwara Y, Tan JH, Adam M (2017). Application of deep convolutional neural network for automated detection of myocardial infarction using ECG signals. Inf. Sci..

[11] Kiranyaz S, Ince T, Gabbouj M (2015). Real-time patient-specific ECG classification by 1-D convolutional neural networks. IEEE Trans. Biomed. Eng..

[12] Zhang F, Li M, Song L, Wu L, Wang B (2023). Multi-classification method of arrhythmia based on multi-scale residual neural network and multi-channel data fusion. Front. Physiol..

[13] Desai U (2016). Diagnosis of multiclass tachycardia beats using recurrence quantification analysis and ensemble classifiers. J. Mech. Med. Biol..

[14] Li Y, Zhang Z, Zhou F, Xing Y, Li J, Liu C (2021). Multi-label classification of arrhythmia for long-term electrocardiogram signals with feature learning. IEEE Trans. Instrum. Meas..

[15] Elgendi M (2012). On the analysis of fingertip photoplethysmogram signals. Curr. Cardiol. Rev..

[16] Allen J (2007). Photoplethysmography and its application in clinical physiological measurement. Physiol. Meas..

[17] Whiting, S., Moreland, S., Costello, J., Colopy, G., & McCann, C. Recognising cardiac abnormalities in wearable device photoplethysmography (PPG) with deep learning. *arXiv Prepr. arXiv1807.04077*, (2018).

[18] Pereira T (2020). Photoplethysmography based atrial fibrillation detection: A review. NPJ Digit. Med..

[19] Sološenko A, Petrėnas A, Marozas V (2015). Photoplethysmography-based method for automatic detection of premature ventricular contractions. IEEE Trans. Biomed. Circuits Syst..

[20] Sološenko, A., Paliakaitė, B., Marozas, V., & Sörnmo, L. Training convolutional neural networks on simulated photoplethysmography data: application to bradycardia and tachycardia detection. *Front. Physiol.* (2022). 10.3389/fphys.2022.928098 PMC9339964 35923223

[21] Cheng P, Chen Z, Li Q, Gong Q, Zhu J, Liang Y (2020). Atrial fibrillation identification with PPG signals using a combination of time-frequency analysis and deep learning. IEEE Access.

[22] Neha N, Sardana H, Kanawade R, Dogra N (2023). Photoplethysmograph based arrhythmia detection using morphological features. Biomed. Signal Process. Control.

[23] Neha K, Sardana H, Dogra N, Kanawade R (2022). Dynamic time warping based arrhythmia detection using photoplethysmography signals. Signal Image Video Process..

[24] Clifford, G. D. *et al. *The PhysioNet/computing in cardiology challenge 2015: reducing false arrhythmia alarms in the ICU. In *2015 Computing in Cardiology Conference (CinC)*, IEEE, pp. 273–276 (2015). 10.1109/CIC.2015.7408639 PMC4910643 27331073

[25] Ababneh, M. Utilization of Photoplethysmography (PPG) Signal Towards Heart Arrhythmia Detection and Classification. Yarmouk University, (2022).

[26] Qananwah Q, Dagamseh A, Alquran H, Ibrahim KS, Alodat M, Hayden O (2020). A comparative study of photoplethysmogram and piezoelectric plethysmogram signals. Phys. Eng. Sci. Med..

[27] Plesinger, F., Klimes, P., Halamek, J., & Jurak, P. False alarms in intensive care unit monitors: detection of life-threatening arrhythmias using elementary algebra, descriptive statistics and fuzzy logic. In *2015 Computing in Cardiology Conference (CinC)*, IEEE, pp. 281–28 (2015).

[28] Fallet, S., Yazdani, S., & Vesin, J. M. A multimodal approach to reduce false arrhythmia alarms in the intensive care unit. In *2015 Computing in Cardiology Conference (CinC)*, IEEE, pp. 277–280 (2015).

[29] Antink, C. H., & Leonhardt, S. Reducing false arrhythmia alarms using robust interval estimation and machine learning. In *2015 Computing in Cardiology Conference (CinC)*, IEEE, pp. 285–288 (2015)

[30] Eerikäinen, L. M., Vanschoren, J., Rooijakkers, M. J., Vullings, R., & Aarts, R. M. Decreasing the false alarm rate of arrhythmias in intensive care using a machine learning approach. In *2015 Computing in Cardiology Conference (CinC)*, IEEE, pp. 293–296 (2015).

[31] Kalidas, V., & Tamil, L. S. Enhancing accuracy of arrhythmia classification by combining logical and machine learning techniques. In *2015 Computing in Cardiology Conference (CinC)*, IEEE, pp. 733–736 (2015).

[32] Caballero, M., & Mirsky, G. M. Reduction of false cardiac arrhythmia alarms through the use of machine learning techniques. In *2015 Computing in Cardiology Conference (CinC)*, IEEE, pp. 1169–1172 (2015).

[33] Paradkar, N., & Chowdhury, S. R. Cardiac arrhythmia detection using photoplethysmography. In *2017 39th Annual International Conference of the IEEE Engineering in Medicine and Biology Society (EMBC)*, IEEE, pp. 113–116 (2017). 10.1109/EMBC.2017.8036775 29059823

